# The spatial stress of urban land expansion on the water environment of the Yangtze River Delta in China

**DOI:** 10.1038/s41598-022-21037-2

**Published:** 2022-10-11

**Authors:** Yufan Chen, Yong Xu, Kan Zhou

**Affiliations:** 1 Key Laboratory of Regional Sustainable Development Modeling, Institute of Geographic Sciences and Natural Resources Research, Chinese Academy of Sciences, Beijing, 100101 China; 2 University of Chinese Academy of Sciences, Beijing, 100049 China

**Keywords:** Environmental sciences, Environmental social sciences

## Abstract

In highly urbanized and industrialized areas, the demand for construction land is expanding, which should have an impact on the water environment. Taking the Yangtze River Delta (YRD) and considering chemical oxygen demand (COD) and ammonia nitrogen (NH_3_-N) as characteristic pollutants, this study investigated the spatial–temporal characteristics of water pollutant emissions at the county level, optimized the spatial lag model (SLM) to estimate the spatial interaction of urban expansion and water pollutant emissions through direct and indirect effects. The results show that from 2011 to 2015, water pollutant emissions in the YRD decreased significantly and that the high-emissions pattern changed from a contiguous to a scattered distribution. The emissions of COD and NH_3_-N in counties at various distances from the Yangtze River and coastline show a logarithmic curve relationship. The association between urban expansion and water pollutant emissions was significant and stable. In 2015, every 1% increase in the scale of urban expansion resulted in 0.299% and 0.340% increases in local COD and NH_3_-N emissions, respectively, and emissions in the adjacent counties synchronously increased by 0.068% and 0.084%, respectively. The results show that to break the association and spatial interaction between urban expansion and water pollutant emissions and alleviate the environmental stress on the YRD, in addition to delimiting an urban expansion boundary and strictly restraining the scale of expansion, improvement in the regional environmental carrying capacity through urban water pollutant treatment facilities and pipe network construction is urgently needed.

## Introduction

The realization of human well-being and sustainable development goals in the process of rapid urbanization has long been threatened by a series of escalating water pollution. Growing cities and land-use changes result in surface hardening of natural areas, reducing infiltration and aquifer recharge, while increasing water run-off and pollution^1^. Urbanization is the process by which a country or region changes from a traditional, rural society to a modern, urban society. It impacts the environment through population movement, economic development and landscape transformation^2,3^. Urban expansion is an important feature of urbanization. Rapid urbanization means a large-scale influx of the rural population into a city, which requires increased impervious surface to meet basic space needs for production and living. As a result, the ecological space becomes occupied and the original structure of the regional environment is changed, causing ecological problems such as urban water environment degradation and biodiversity reduction^4–6^. Urban expansion is accompanied by high-intensity and high-density production and living activities. Many pollutants and dangerous chemicals continue to leak or dump in urban areas and surrounding waters, which inevitably leads to the deterioration of human settlements and even endangers human well-being^7,8^. To make the rapidly expanding urban areas more sustainable, it is urgent to curb the threat of various types of water pollutants to the regional water ecosystem. Every city should pay an active role in urbanization and environmental change, thus to create a healthier life and well-being for urban population^1,9,10^.

Research on the environmental effects of urban expansion has mainly focused on the atmospheric environment. In general, common pollutants such as sulfur dioxide, ozone and particulate matter have often been researched, and the relationship between air pollution and urban construction land expansion has been quantitatively analyzed by using GIS and regression analysis methods^11–13^. The aerosol characteristics retrieved from satellite remote sensing data have been used to analyze the urban air pollution caused by various human factors such as industrial activities, traffic pollution and engineering construction^14–16^. The results of these studies have shown that the distribution of urban land determines the spatial pattern of air pollutant emission sources^17–19^, and construction land, as the dominant factor, is positively correlated with urban air pollution^20–22^.

Research on water environments and urban expansion has mainly obtained the data of water pollutant emissions through sampling, monitoring and simulation experiments and then deconstructed the interaction mechanism between urban expansion and water pollution by using spatial measurement, path analysis, redundancy analysis and other methods^23–25^. Empirical studies have shown that urban land use structure has a spatial correlation with most water pollutant emissions^26–28^. The expansion of non-agricultural land and the contraction of arable land affect the intensity of water pollutant emissions to varying degrees, increasing the loads of ammonia nitrogen (NH_3_-N), organic matter, heavy metals and other pollutants in the water of industrial and urban living areas^29,30^.

Some studies have revealed that environmental pollution comprises not only point-to-point pollution but also diffuse spatial spillover pollution^31^. Through many long time-series and multi-scale studies, scholars have confirmed that there are significant spatial correlation characteristics of water pollution and that adjacent basins often show similar water pollution patterns or change characteristics^32–35^. Urbanization is an important driving force of the coordinated development of urban agglomerations. Different urbanization processes result in different environmental pollution characteristics^36,37^. Compared with other forms of pollution, air pollution is more easily perceived^38^. With the support of geographically weighted regression, threshold models, the spatial Durbin model and other spatial econometric models, empirical studies have shown a positive correlation between urbanization and air pollution^39^. Especially in regional carbon emission and haze pollution, urbanization as the dominant factor has a significant spatial spillover effect^40,41^. That is, an increase in local urbanization exacerbates the degree of environmental pollution in adjacent areas, and there are cross effects between adjacent regions^42,43^.

Based on the research, we find that although the acceleration of urbanization and the expansion of urban scale promote economic development, they also increase the load on urban water resources and the water environment. It is important to understand the associated effect of urban expansion on water pollutant emissions and its spatial interaction mechanism. Studies have failed to quantify the intensity of this effect and the spatial differentiation of pollutant emissions caused by urban land expansion by using multiple scale analysis. Including construction land with rural construction land, transportation facilities land and other types makes it difficult to accurately estimate the impact of urban expansion. The regional pollution effect caused by a single city in urban agglomeration has yet to be analyzed from spatial dimension.

This study considers the case of the Yangtze River Delta (YRD) and integrates multi-source data, such as water pollutant emissions, land use, population and economy, to depict the temporal and spatial variation characteristics of water pollutant emissions at the county level from 2010 to 2015. A spatial econometric model is used to quantitatively analyze the associated effect and spatial mechanism of urban expansion and water pollutant emissions. This paper focuses on the following issues: (1) how to quantify the local and regional water pollution emissions caused by individual urban expansion; (2) how urban expansion affects water pollutant emissions and whether there is a spatial spillover effect and (3) how to effectively reduce the negative spillover effect of urban expansion and achieve high-quality environmental development in urban agglomerations, particularly areas with rapid urbanization and large-scale populations and economies.

## Methods and data

### Study area and data source

#### Study area

**Figure 1:**
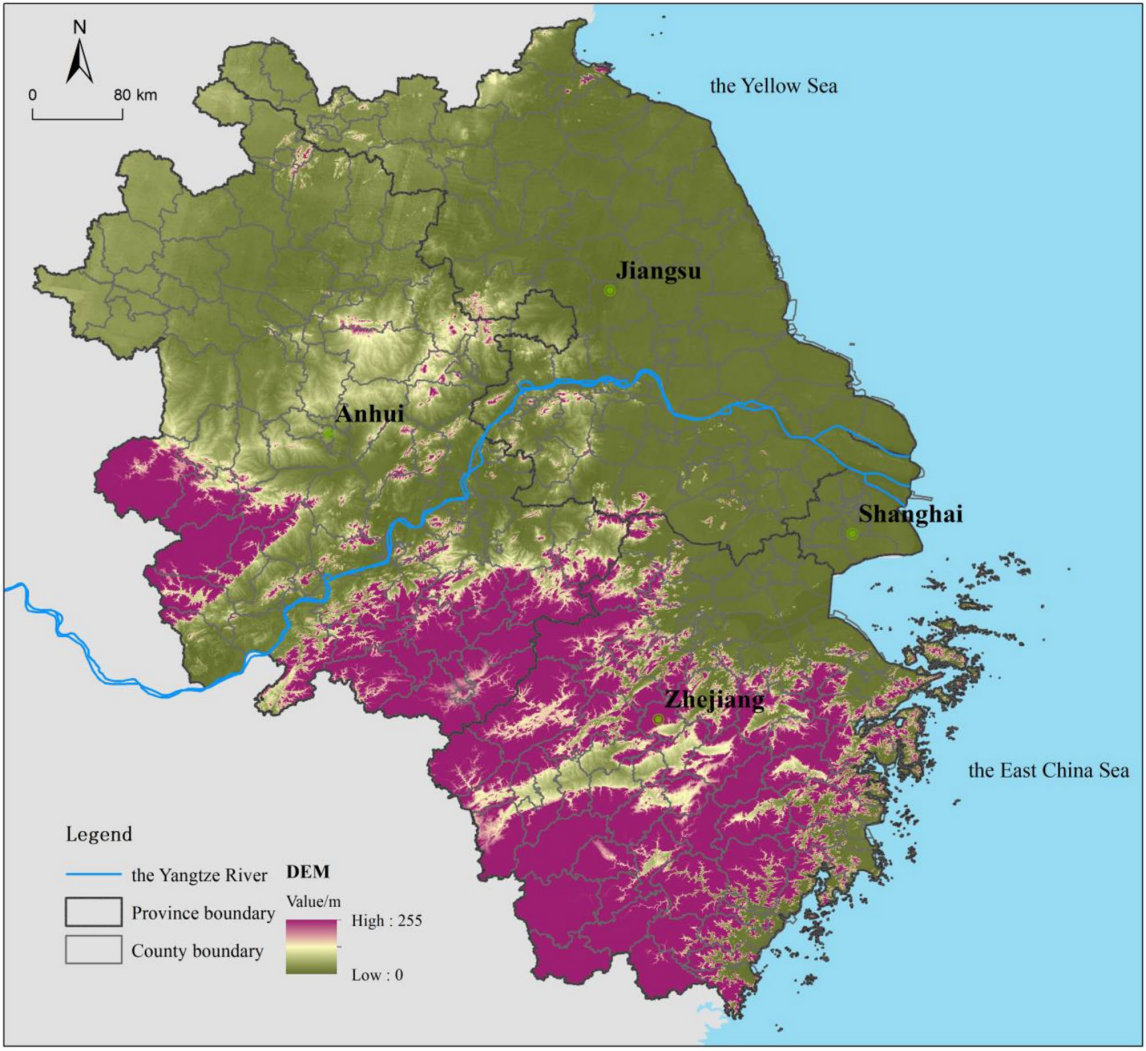
Location of the Yangtze River Delta. Map was generated by ArcGIS 10.2 (http://www.esri.com/software/arcgis/arcgis-for-desktop).

The YRD is located on the east coast of China and includes Shanghai city, Jiangsu Province, Zhejiang Province and Anhui Province (Fig. 1), with a total area of 358,000 km^2^. The YRD is one of the most powerful regions in China, leading the economic development of the Yangtze River Economic Belt and that of the country. In 2015, the resident population of the YRD was 221 million (16.06% of China’s population), and its GDP was 16.01 trillion yuan (23.36% of China’s GDP). However, the YRD’s rapid urban expansion and high-intensity resource and energy consumption have long produced high water pollutant emissions and demonstrated high per-unit emissions intensity. The YRD only accounts for 4% of China’s land but produces 21.21% of the country’s wastewater emissions. Rapid urbanization and industrialization in the YRD have resulted in vast environmental pollution in rivers, lakes and seas; consequently, the regional water ecological imbalance is serious, and in some areas, even threatens the safety of the drinking water^44^.

#### Data selection

Using the data of 305 county-level administrative regions in the YRD, including counties, municipal districts and county-level cities, as the basic statistical units, we established a database of land use, water pollutant emissions and economic development from 2010 to 2015. The specific data sources are as follows.

Administrative division data at the country, province, city and county levels were obtained from the website of the National Geomatics Center of the People’s Republic of China (PRC). The land use data at the county level were collected from the Ministry of Natural Resources of the PRC. The pollutant emission and socioeconomic data were obtained from the China County Statistical Yearbook, Shanghai Statistical Yearbook, Jiangsu Statistical Yearbook, Zhejiang Statistical Yearbook and Anhui Statistical Yearbook. Local city statistical yearbooks or county statistical bulletins were used to provide the incomplete or missing data of 33 counties.

### Spatial econometric model

#### Spatial correlation analysis

Due to Tobler's first law of geography, most spatial data has a strong or weak spatial correlation. Moran’s I statistic has commonly been used to test the global and local spatial autocorrelations among water pollution variables. The global Moran’s I statistic can be calculated as follows:

1 $${I}_{Global}=\frac{N{\sum }_{i}\sum_{j}{\omega }_{ij}({x}_{i}-\overline{x })({x}_{j}-\overline{x })}{{\sum }_{i}\sum_{j}{\omega }_{ij}\sum_{i}{({x}_{i}-\overline{x })}^{2}},$$

where $N$ is the total number of spatial units, ${\omega }_{ij}$ is the spatial weight matrix, ${x}_{i}$ and ${x}_{j}$ are the water pollutant emissions of county $i$ and $j$, respectively, $\overline{x }$ is the average water pollutant emissions. The global Moran’s I has a value in the range of $\left[-1,1\right]$.

The local distribution of spatial elements among regions may be atypical, which cannot be reflected by global indicators. The local Moran’s I statistic can be used to test the spatial correlation between counties as follows:

2 $${I}_{Local}=\frac{({x}_{i}-\overline{x })}{\left[\sum_{j=1}^{N}{\omega }_{ij}{({x}_{j}-\overline{x })}^{2}/(N-1)\right]-\overline{{x }^{2}}}\times \sum_{j=1}^{N}{\omega }_{ij}\left({x}_{j}-\overline{x }\right),$$

#### Parameter estimation model of the spatial effect

According to the theoretical analysis and characteristics of the spatial mobility of water pollution, water pollutant emission in urban expansion has a spatial effect; thus, we constructed a spatial econometric model for parameter estimation. First, the SLM and spatial error model (SEM) were constructed. Then, the optimal model was selected according to the Lagrange multiplier (LM) test and spatial effect decomposition requirements^45,46^. The expressions of each model are as follows.

1. *SLM* When there is an endogenous interaction effect, the spatial lag term of the explained variable must be added to the general linear regression model, transforming it into SLM.
  3 $$Y=\alpha {I}_{N}+\rho WY+\beta X+\varepsilon ; \varepsilon -N\left(0,{\delta }^{2}{I}_{N}\right).$$
2. *SEM* When there is an interaction effect of an error term, that is, when there is a spatial autocorrelation of a model error term, it is necessary to add a spatial correlation error term and transform it into SEM.
  4 $$Y=\alpha {I}_{N}+\beta X+\lambda W\mu +\varepsilon ; \varepsilon -N\left(0,{\delta }^{2}{I}_{N}\right).$$
  where $Y$ is the explained variable, $X$ is the exogenous explanatory variable matrix, ${I}_{N}$ is the unit vector,$W$ is the spatial weight matrix, $\rho$ is the spatial autoregressive coefficient of the explained variable,$\alpha$ is a constant, $\beta$ is the regression coefficient vector of the explanatory variable, $\lambda$ is the spatial autocorrelation coefficient between the regression residuals and $\varepsilon$ is the unexplained random error term.

#### Measurement model of the interaction mechanism

In view of the influences of exogenous explanatory variables on the local explained variable, i.e., direct effects, and the influences of exogenous explanatory variables on other surrounding explained variables, i.e., indirect or spatial spillover effects^47,48^, the total spatial effect can be decomposed using the partial differential method, and the SLM can be converted into the following:

5 $$Y = \left( {1 - \rho W} \right)^{ - 1} \alpha I_{N} + 1 - \rho W^{ - 1} \beta X + \left( {1 - \rho W} \right)^{ - 1} \varepsilon .$$

Next, the partial derivative of each explanatory variable can be calculated as follows.

6 $$\left[ {\frac{\partial Y}{{\partial X_{1K} }}, \ldots ,\frac{\partial Y}{{\partial X_{NK} }}} \right] = \left[ {\begin{array}{*{20}c} {\frac{{\partial Y_{1} }}{{\partial X_{1K} }}} & \ldots & {\frac{{\partial Y_{1} }}{{\partial X_{NK} }}} \\ \vdots & \ddots & \vdots \\ {\frac{{\partial Y_{N} }}{{\partial X_{1K} }}} & \ldots & {\frac{{\partial Y_{N} }}{{\partial X_{NK} }}} \\ \end{array} } \right] = I - \rho W^{ - 1} \left[ {\begin{array}{*{20}c} {\begin{array}{*{20}c} {\gamma 1} & {\beta_{12} } \\ {\beta_{21} } & {\gamma 2} \\ \end{array} } & {\begin{array}{*{20}c} \ldots & {\beta_{1N} } \\ \ldots & {\beta_{2N} } \\ \end{array} } \\ {\begin{array}{*{20}c} \vdots & \vdots \\ {\beta_{N1} } & {\beta_{N2} } \\ \end{array} } & {\begin{array}{*{20}c} \ddots & \vdots \\ \ldots & {\gamma K} \\ \end{array} } \\ \end{array} } \right],$$

where the average value of the diagonal coefficient represents the direct effect, reflecting the actual influence of the explanatory variables on the local explained variable, whereas the average value of the nondiagonal coefficient represents the indirect effect, representing the average influence of the explanatory variables on the explained variable of the surrounding regions.

### Indicator selection and variable descriptions

#### Explained variables

Chemical oxygen demand (*COD*) and *NH*_3_*-N* were the explained variables. These two pollutants are listed as the main emission control indicators by the environmental authorities of the central government and YRD, providing a certain time continuity in the statistics. Furthermore, COD and NH_3_-N in the YRD account for 12.63% and 16.33%, respectively, of the total amounts of these pollutants in the country. They are also the main control targets of water pollutant emissions reduction efforts in the YRD.

#### Core explanatory variable

Urban expansion is the process of increasing the scale of land used for urban development and functional operations; it is the inevitable result of rapid urbanization and urban modernization. In this paper, the urban expansion scale (*UES*) was the core explanatory variable, and it was obtained by summing the area of various types of land used for urban construction and industrial and mining activities in each county.

#### Control variables

For robustness, other economic and social development indicators that may affect the emission of water pollutants were selected as control variables. As shown in Table 1, *POP* is the number of permanent residents and is used to describe the scale of urban population. *PGDP* is per capita GDP, which represents the level of urban economic development. *IS* is the ratio of the added value of the secondary industry to GDP, i.e., the industrial structure and level of urban industrialization. *FDI* is the amount of foreign direct investment, which is used to characterize the openness of the urban economic market. *FAI* is the total investment in fixed assets, which indicates the scale of domestic capital investment and asset reproduction capacity. *FD* is the ratio of local general budget revenues to general budget expenditures, which reflects the financial autonomy of the region. Furthermore, to analyze the possible effect of geographical location on water pollutant emissions, the following spatial variables were set; *Dist-coast*, expressed as the shortest distance between the county and the coastline of the East China Sea, and *Dist-yangtze*, expressed as the shortest distance between the county and the Yangtze River mainstream.

**Table 1:** Descriptive statistics of the main variables.

Variables	Description (unit)	Year	Mean	SD	Max	Min
COD	Chemical oxygen demand emission (tons)	2010	10,328.42	7548.85	52,562.64	920.18
		2015	6056.58	4354.81	33,281.90	348.49
NH_3_-N	Ammonia nitrogen emission (tons)	2010	1373.67	1014.05	11,083.43	111.98
		2015	885.50	749.60	9286.15	20.33
UES	Urban expansion scale (km^2^)	2010	86,434.45	73,460.95	731,127.00	5689.20
		2015	92,483.36	76,296.67	756,861.00	6802.35
POP	Permanent population (ten thousand people)	2010	66.89	40.92	278.53	7.66
		2015	67.43	41.49	288.44	7.75
PGDP	Per capita GDP (RMB yuan/person)	2010	55,654.09	48,624.38	345,549.67	4574.97
		2015	76,902.10	69,024.62	565,437.42	6561.63
IS	Industrialization level (%)	2010	48.86	14.67	81.98	5.50
		2015	45.23	13.76	79.93	3.31
FDI	Foreign direct investment (ten thousand US dollars)	2010	206,283.70	307,276.08	1,260,055.00	4430.00
		2015	249,685.73	415,456.78	1,845,923.00	6006.00
FAI	The total investment in fixed assets (billion yuan)	2010	147.86	147.41	1435.39	4.04
		2015	305.59	233.68	1772.94	14.80
FD	The local financial autonomy (%)	2010	0.73	0.40	2.89	0.09
		2015	0.72	0.38	2.58	0.13

As the core variable, *UES* has a strong positive correlation with COD and NH_3_-N emission. The person correlation coefficients were 0.503 and 0.573, respectively, and they all passed the significance test at 1% level. Control variables were correlated with COD and NH_3_-N emissions in varying degrees, among which *POP* was a strong correlation, and the other variables were weak or very weak correlation (Supplementary Appendix Table 1). Furthermore, the average VIF of all variables was 2.56, and the individual value was less than 10, which indicates that there is no multicollinearity among explanatory variables and does not affect the spatial econometric analysis.

## Results

### Temporal and spatial characteristics of water pollutant emissions

During 2010–2015, the scale of water pollutant emissions in the YRD decreased significantly (Supplementary Appendix Table 2). Specifically, COD emissions decreased 41.36% from 3.15 million tons to 1.85 million tons, and total NH_3_-N emissions decreased 35.54% from 41.90 million tons to 27.01 million tons. The intensity of water pollutant emissions in counties also showed a downward trend. In 2010, the average emissions of COD and NH_3_-N in a county were 10,328.42 tons and 1373.67 tons, respectively. By 2015, they had decreased to 6065.58 tons and 885.50 tons, respectively. A comparison of the emissions of various provinces revealed that COD and NH_3_-N emissions in Jiangsu Province accounted for about 40% of the total amount in the YRD in the 5-year span. The rates of emissions reduction for these two pollutants were 34.93% and 34.11%, respectively. Thus, Jiangsu Province provided a large share of the YRD’s water pollutant emissions, and its emission reduction effects were also more prominent.

**Figure 2:**
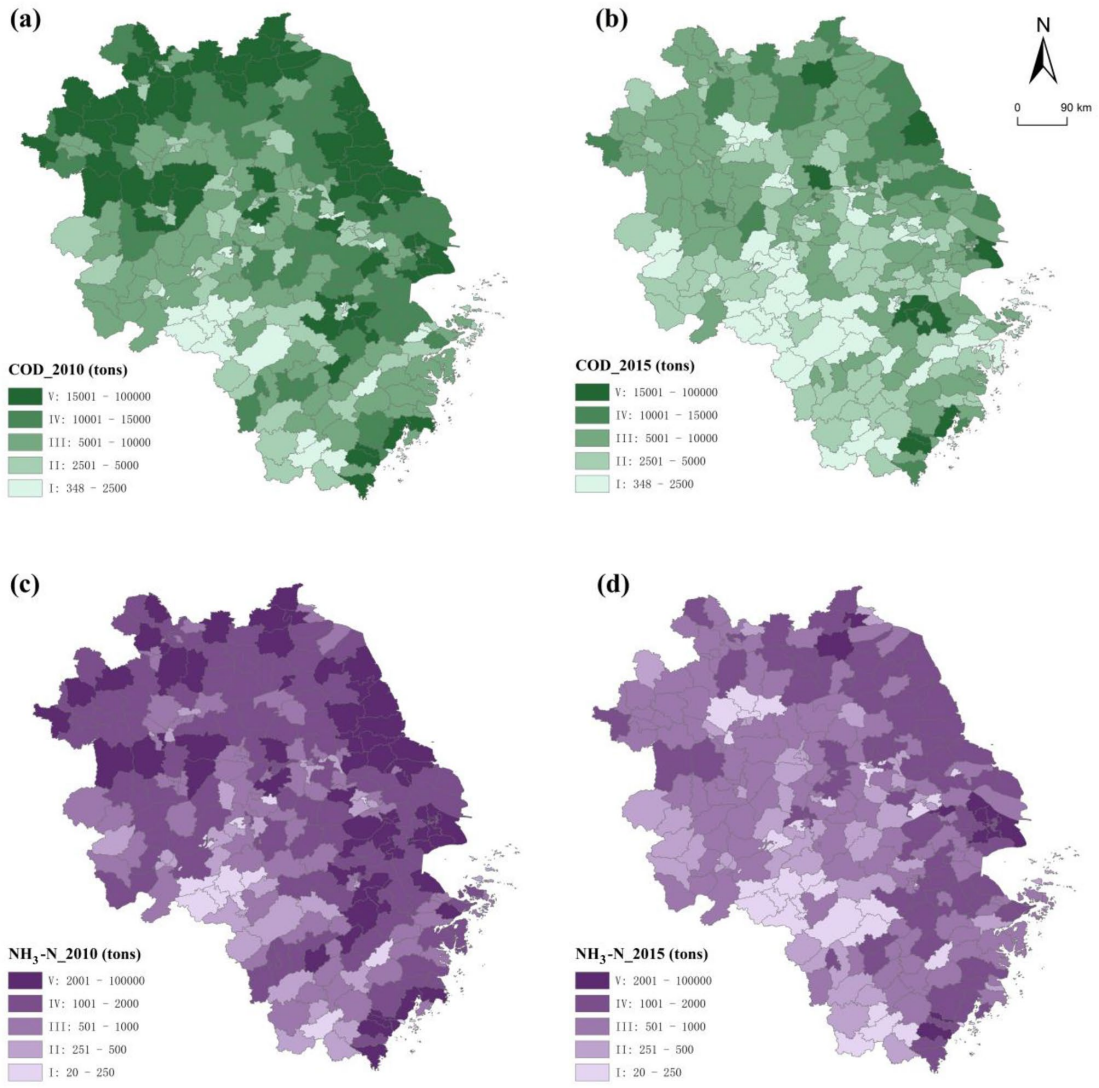
Classification of counties by COD (**a,b**) and NH_3_-N (**c,d**) emissions. Map was generated by ArcGIS 10.2 (http://www.esri.com/software/arcgis/arcgis-for-desktop).

The spatial distribution of water pollutant emissions by counties in the YRD is shown in Fig. 2. It can be seen from Table 2 that the number of counties whose COD emissions decreased by 1, 2, 3, and 4 levels are 130, 55, 2 and 1 respectively. Among them, the number of counties with COD emission grades of V and IV, i.e., emission intensities of more than 10,000 tons, decreased from 128 to 43 specially (Fig. 2a,b). Similarly, the NH_3_-N emissions of 137, 30 and 2 counties decrease by 1, 2 and 3 levels respectively, and the number of counties with NH_3_-N emission grades of V and IV decreased from 179 to 100 (Fig. 2c,d). Comparing these four subfigures, we can see that no matter what kind of pollutant, the distribution of V-class counties shrank from the large-scale, continuous distribution in Shanghai; provincial capital cities, such as Hangzhou, Hefei and Nanjing; the eastern coastal area and the Northern region in 2010, forming a scattered distribution pattern in 2015. Emission intensity at the county level increased from the central urban area throughout the surrounding area. This shows that urban expansion is spatially related to water pollution emission levels. In addition, the emission intensities of counties near provincial or city administrative boundaries were higher than those of counties within cities.

**Table 2:** Changes in pollutant emission levels.

	4 Levels	3 Levels		2 Levels			1 Level			
	V–I	V–II	IV–I	V–III	IV–II	III–I	V–IV	IV–III	III–II	II–I
COD	1	1	1	33	13	9	18	42	44	26
NH_3_-N	0	0	1	17	8	5	39	58	26	14

**Figure 3:**
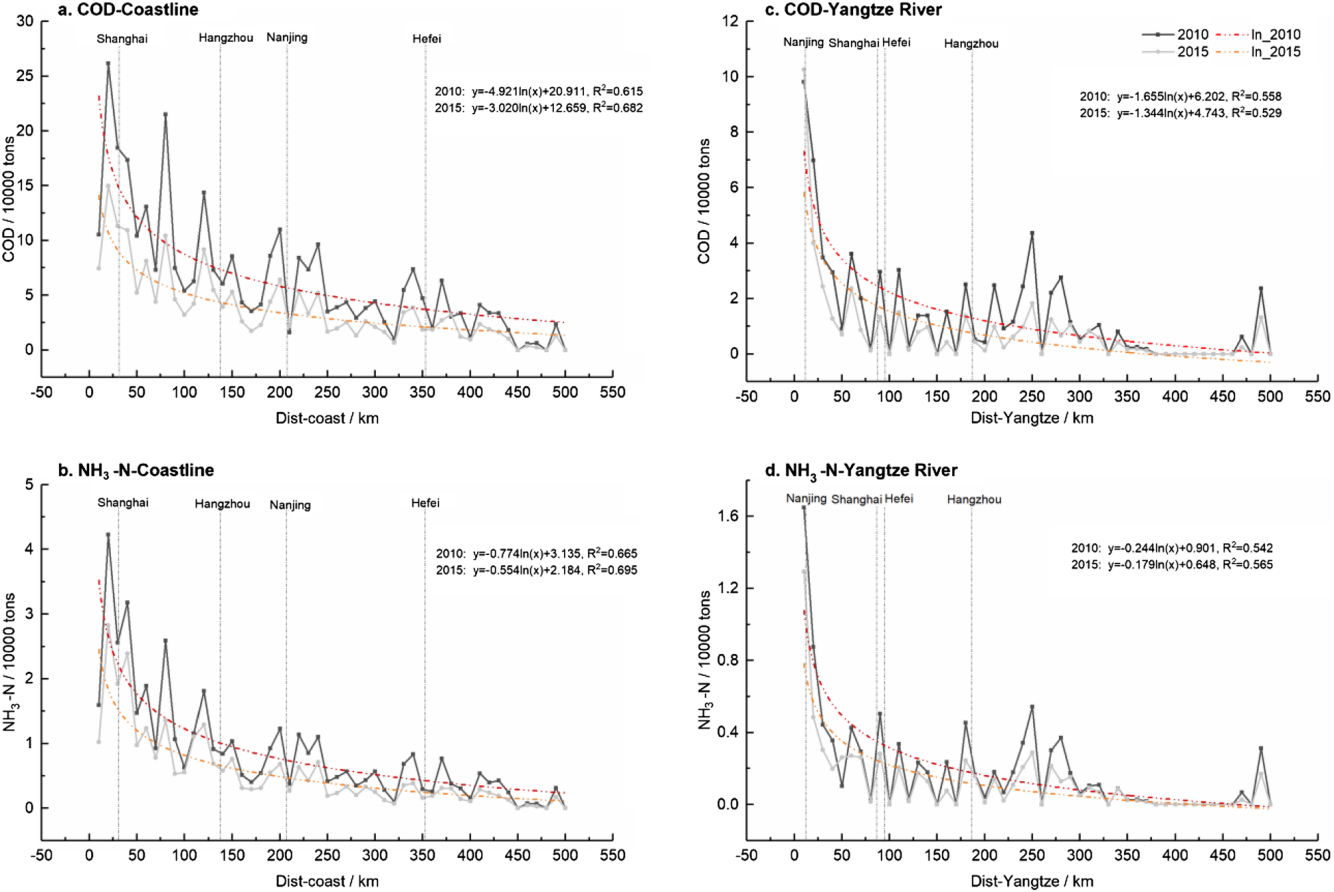
Water pollutant emissions of counties by distance from coastline or the Yangtze River mainstream.

Taking 10 km as the buffer distance, the COD and NH_3_-N emissions of counties were measured at different distances from both the coastline and the main stream of the Yangtze River. To avoid possible interference from coastal locations, counties within 100 km of the coastline were not included. As shown in Figs. 3, the emissions of COD and NH_3_-N in counties at various distances show a logarithmic curve relationship, and the overall emissions in 2015 were lower than in 2010. Specifically, it can be seen from Fig. 3a,b that the water pollutant emissions of coastal counties were higher than those of inland counties, and COD and NH_3_-N emissions of counties within 100 km of the coastline accounted for 43.69% and 50.34% of total YRD emissions, respectively. Moreover, because nearly half of the counties in Shanghai, Zhejiang, and Jiangsu are close to the East Sea, their overall pollution emission intensity will be higher than that of Anhui. Similarly, the COD and NH_3_-N emissions of counties within 50 km of the Yangtze River mainstream accounted for 47.28% and 42.27% of total YRD emissions, respectively (Fig. 3c,d). The Yangtze River directly passes through Anhui and Jiangsu, and then flows out to Shanghai. Therefore, considering the actual distance between each county and the Yangtze River, the water pollution emissions of Anhui and Jiangsu will be more easily affected by the Yangtze River than that of Zhejiang. Especially in the counties on both sides of the Yangtze River, this degree of influence is more obvious.

### Spatial correlation and agglomeration of water pollutant emissions

**Figure 4:**
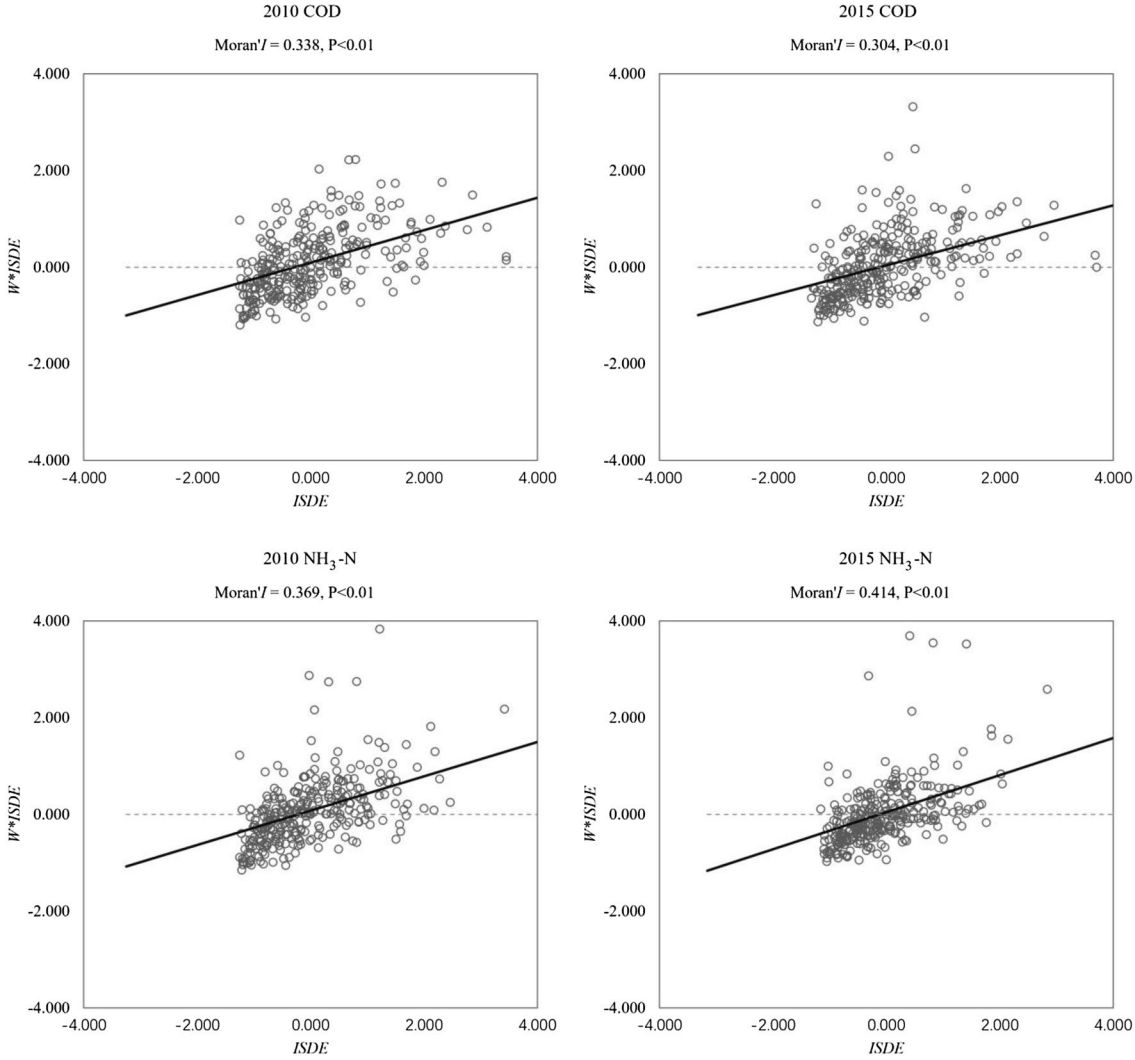
The results of global and local Moran’s I.

The global Moran’s I of water pollutant emissions is significantly positive (P < 0.01), which shows that there is a positive spatial correlation of water pollutant emissions in the YRD. As shown in Fig. 4, the emission of COD and NH_3_-N both show strong spatial agglomeration. The global Moran’s I of COD emissions decreased from 0.338 in 2010 to 0.304 in 2015, while that of NH_3_-N emissions increased from 0.369 to 0.414 on the contrary. It means that the spatial agglomeration of COD emissions has been weakened, while that of NH_3_-N emissions has been enhanced from 2010 to 2015.

**Figure 5:**
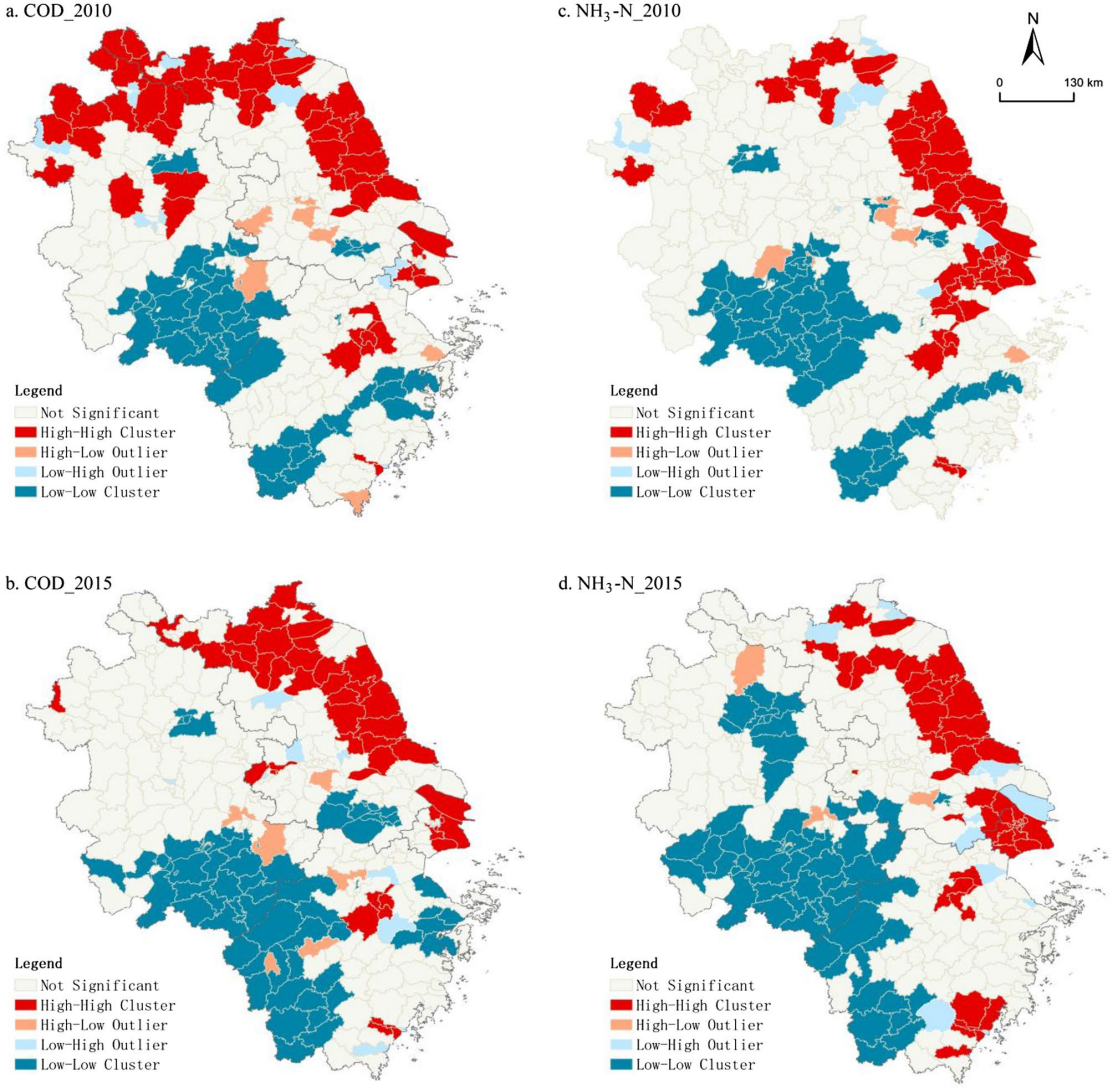
Local spatial agglomeration of water pollutant emissions in the YRD. Map was generated by ArcGIS 10.2 (http://www.esri.com/software/arcgis/arcgis-for-desktop).

We can further get four types of spatial agglomeration, namely high-high (HH), high-low (HL), low–high (LH) and low-low (LL) cluster. As shown in Fig. 5a,b, the HH cluster of COD emissions disappeared from northern Anhui in 2015, and more concentrated in northeast Jiangsu and eastern Shanghai; the LL cluster of COD emissions gradually expanded from southern Anhui to western Zhejiang and southern Jiangsu. Due to industrialization and urbanization, the eastern coastal areas are prone to form large-scale industrial clusters and population agglomerations, thus forming a contiguous pattern of high pollution in space. However, due to the influence of natural and geographical factors, the western Zhejiang region belongs to the ecological region, so it is more characterized by low pollution agglomeration. It also can be seen from Fig. 5c,d that the HH cluster of NH_3_-N emissions has not changed greatly, mainly concentrated in northeast Jiangsu and Shanghai, while the LL cluster of NH_3_-N expanded to central Anhui and western Zhejiang. The emission of NH_3_-N is more related to daily life. The overall change in the spatial distribution of population from 2010 to 2015 is smaller than that of the industrial pattern, so the pollution pattern is relatively stable.

### Estimation of spatial effects

The spatial regression results of the SLMs are shown in Table 3. The estimated parameters of SLMs (1), (3), (5) and (7) all show that the associated effects of urban expansion and water pollutant emissions were quite significant, and *UES* was the dominant factor affecting water pollutant emissions in the YRD. In 2010, each 1% increase in *UES* in the YRD increased COD and NH_3_-N emissions by 0.324% and 0.297%, respectively. By 2015, however, the associated effect of *UES* on COD emission decreased, the associated effect of *UES* on NH_3_-N emission increased, and their coefficients were 0.293 and 0.335, respectively. During rapid urbanization in the YRD, large-scale urban expansion translated into a rapid increase in the spatial diffusion of urban production and living functions, which directly promoted the inflow of economic and social factors into the cities, expanded the scale of industrial production capacity and living activities in urbanized areas and promoted high emissions of urban domestic and industrial water pollutants.

**Table 3:** Estimation results for associated effects based on contiguity edges matrix.

Variables	COD_2010		COD_2015		NH_3_-N_2010		NH_3_-N_2015	
	SLM (1)	SLM (2)	SLM (3)	SLM (4)	SLM (5)	SLM (6)	SLM (7)	SLM (8)
C	0.719	1.726*	0.491	1.139	− 1.026	0.465	− 2.286**	− 0.409
ln UES	0.324***	0.326***	0.293***	0.296***	0.297***	0.300***	0.335***	0.353***
ln POP	0.740***	0.700***	0.571***	0.580***	0.760***	0.722***	0.622***	0.581***
ln PGDP	0.077	0.008	0.242***	0.225***	0.169***	0.079	0.356***	0.254***
ln IS	0.189***	0.189***	− 0.114	− 0.119	− 0.091*	− 0.093*	− 0.263***	− 0.276***
ln FDI	− 0.105***	− 0.094***	− 0.166***	− 0.171***	− 0.084***	− 0.071***	− 0.125***	− 0.115***
ln FAI	0.040	0.063	0.067	0.058	− 0.019	0.004	− 0.053	− 0.037
ln FD	− 0.117	− 0.125*	− 0.192**	− 0.225**	0.016	0.004	− 0.090	− 0.106
ln Dist-coast		− 0.066**		− 0.039		− 0.094***		− 0.119***
ln Dist-yangtze		0.003		− 0.046*		− 0.010		− 0.036
*ρ*	0.120***	0.109***	0.191***	0.187***	0.179***	0.151***	0.204***	0.168***
*Sigma^2*	0.177	0.173	0.246	0.243	0.134	0.128	0.246	0.237
*R*^*2*^	0.707	0.712	0.562	0.564	0.759	0.769	0.597	0.610
*LLF*	− 169.641	− 166.098	− 220.126	− 218.390	− 127.996	− 120.148	− 220.462	− 214.042

***P < 0.01, **P < 0.05 and *P < 0.1.

The spatial autoregressive coefficients of the eight SLMs were significantly positive at the 1% level, which indicates there were spatial agglomeration and positive spatial spillover effects of water pollutant emissions in the YRD; that is, the increase of local water pollutant emissions may have directly led to the synchronous increase in adjacent counties. The driving effect of other economic control variables on water pollutant emissions showed that *POP*, *PGDP* and *FAI* were positive drivers, whereas *IS*, *FDI* and *FD* were negative drivers. It is also worth noting that the driving intensity of each control variable on water pollutant emissions rose between 2010 and 2015, which means that the regional diffusion of water pollutants became increasingly serious.

After we added the location variables, the estimated results of SLMs (2), (4), (6) and (8) showed negative regression coefficients for *Dist-coast* and *Dist-yangtze* negative; that is, the shorter the distance from the coastline or mainstream of the Yangtze River to the county, the greater the water pollutant emissions of the county. Combined with the statistical analysis of COD and NH_3_-N emissions in the previous section, this shows that coastal and riverside locations were usually chosen for urban expansion and that the production and living activities within 100 km of the coast and within 50 km of the river caused high-intensity water pollutant emissions.

### Spatial effect decomposition

According to the results of the SLMs, the spatial spillover effects of urban expansion and water pollutant emissions were significant, but the estimated coefficients of the SLMs do not fully reflect the spatial interaction mechanism between them. The direct and indirect effects of the variables must be decomposed to further measure the strength of the variables’ impacts.

As shown in Table 4, the direct and indirect effects of the *UES* on COD and NH_3_-N emissions were significant, indicating that urban expansion has not only a direct impact on local water pollutant emissions but also an indirect effect on the emissions of adjacent areas through spatial spillover. The regression coefficient of the direct and indirect effects of *UES* on COD and NH_3_-N emissions were 0.299 and 0.068, respectively, and significant at the 1% level. This shows that every 1% increase in *UES*, the COD emissions of local and adjacent counties increased by 0.299% and 0.068%, respectively. Similarly, urban expansion also aggravated the NH_3_-N emissions in local and adjacent counties, and the coefficients of *UES* were 0.340 and 0.084, respectively.

**Table 4:** Decomposition results for the spatial effects based on contiguity edges matrix.

Explanatory variables	COD_2015			NH_3_-N_2015		
	Direct effect	Indirect effect	Total effect	Direct effect	Indirect effect	Total effect
ln UES	0.299***	0.068***	0.366***	0.340***	0.084***	0.424***
ln POP	0.571***	0.130***	0.701***	0.629***	0.155***	0.784***
ln PGDP	0.243***	0.056***	0.299***	0.366***	0.091***	0.456***
ln IS	− 0.118*	− 0.027*	− 0.145*	− 0.266***	− 0.066***	− 0.332***
ln FDI	− 0.166***	− 0.038***	− 0.204***	− 0.127***	− 0.032***	− 0.158***
ln FAI	0.068	0.016	0.083	− 0.061	− 0.015	− 0.076
ln FD	− 0.201**	− 0.046**	− 0.247**	− 0.095	− 0.024	− 0.119

***P < 0.01, **P < 0.05 and *P < 0.1.

We found three types of spatial interaction between the main control variables and water pollutant emissions:

- Both direct and indirect effects were positive interactions, as was seen with *UES*, *POP* and *PGDP*, meaning that all of these variables simultaneously increased the emissions of water pollutants in local and adjacent counties.
- Both direct and indirect effects were negative interactions, as was seen with *IS*, *FDI* and *FD*, showing that they reduced the water pollutant emissions in local and adjacent counties.
- Spatial interaction was not fixed due by the type of water pollutant, as was seen with *FAI*; rather, it increased the COD emission of local and adjacent counties but reduced their NH_3_-N emissions.

### Robustness test

#### Robustness of SLM

The choice of the analysis model largely determines the robustness of the results. We performed Ordinary Least Squares (OLS) regression analysis and Lagrange Multiplier test (LM) in Geoda software. The results in Table 5 show that Moran’s I (error) strongly rejected the null hypothesis that residuals do not have spatial dependence, indicating that subsequent research must eliminate the spatial dependence factors in the residuals after OLS regression. According to the results of the robust LM test, the adjoint probabilities of Robust LM-lag and Robust LM-error also rejected the respective null hypothesis at the 10% significance level, indicating that SLM and SEM passed the robustness test and were both applicable. However, there is no indirect effect in the regression results of the SEM, which is not conducive to an analysis of the spatial interaction of urban expansion. Therefore, we used the SLM to explore the associated effects of urban expansion on water pollutant emissions.

**Table 5:** Test results of the spatial econometric models.

Test	2010		2015	
	COD	NH_3_-N	COD	NH_3_-N
Moran’s I(error)	0.358***	0.418***	0.289***	0.222***
LM-lag	15.679***	31.937***	27.791***	22.660***
Robust LM-lag	0.200	1.657*	3.950*	3.601*
LM-error	97.690***	133.581***	63.742***	37.653***
Robust LM-error	82.211***	103.301***	39.901***	18.594***

***P < 0.01, **P < 0.05 and *P < 0.1.

**Figure 6:**
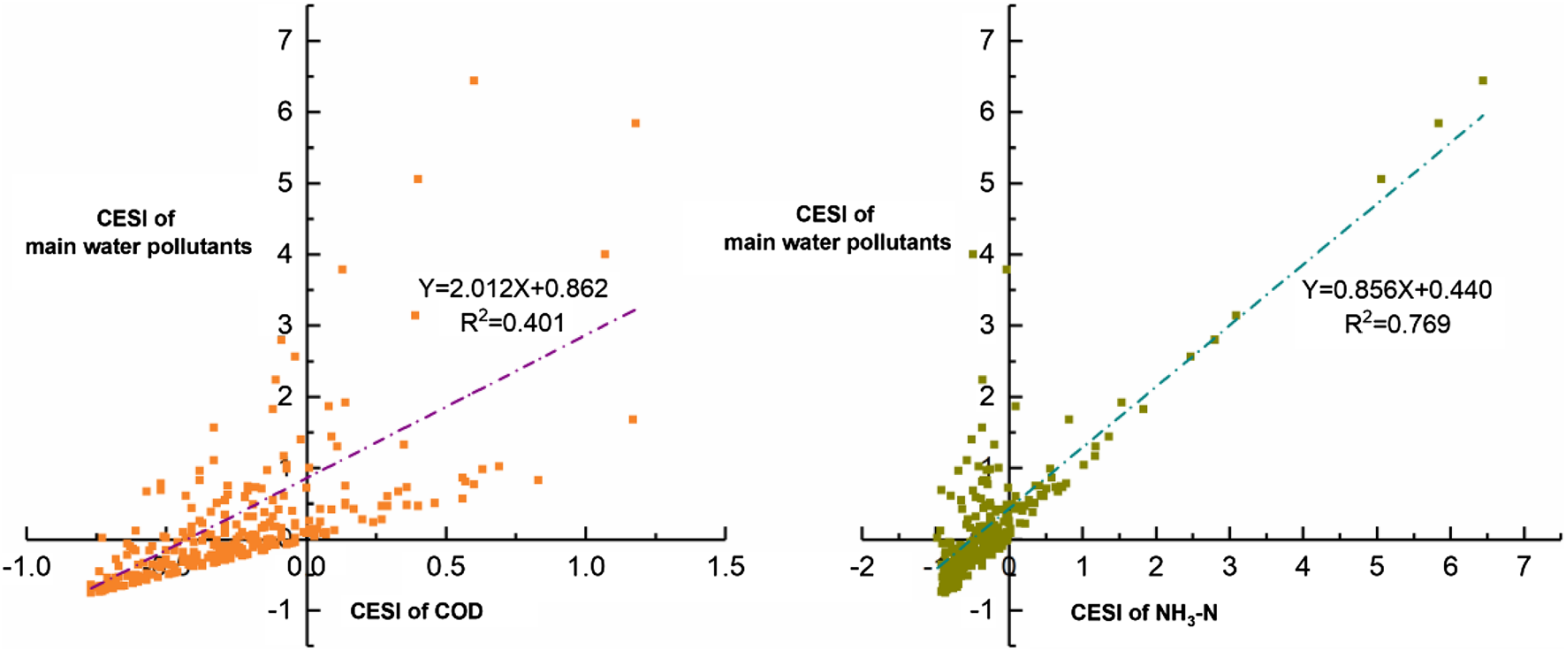
Scatter plot of the CESI of particular pollutants and main water pollutants.

The typicality and prominence of the selected pollutants in the YRD water pollution also affect the robustness of the results. We calculated the water pollutant concentration exceeding the standard index (CESI) for a river or lake reservoir section in the YRD to ensure that the selected indicators are characteristic pollutants that accurately reflect the water pollution^49,50^. The overall concentrations of COD and NH_3_-N in the YRD critically exceeded the standard limits (Supplementary Appendix Table 3). The percentage of counties in the study with above-standard concentrations of COD and NH_3_-N were 18.93% and 16.43%, respectively, and the percentage of counties that critically exceeded limits were 35.71% and 25.36%, respectively. Then, we calculated the overall CESI of the main water pollutants in each county, including dissolved oxygen (OD), COD, NH_3_-N, biochemical oxygen demand (BOD), total nitrogen (TN) and total phosphorus (TP). A scatter plot (Fig. 6) of the CESI of COD, NH_3_-N and main water pollutants showed a strong correlation. The result showed that COD and NH_3_-N were characteristic pollutants capable of accurately reflecting water pollution in the YRD and thus appropriate for use as explained variables for further analysis.

We also tested the robustness of SLM by recalculating the associated effects of *USE* and water pollutant emissions under the inverse distance matrix. The spatial correlation coefficients of *USE* were 0.327, 0.307, 0.303 and 0.347, respectively. Compared with the results based on contiguity edges matrix, the difference could be controlled within 5% (see Supplementary Appendix Table 4). Similarly, we decomposed the spatial effects again under the new spatial matrix, and the results showed that the direct and indirect effect of *USE* were still in the error range. Furthermore, the re calculated correlation coefficients of *USE* all passed the significance test, and the significance level did not change, which further illustrated the applicability and robustness of SLM (see Supplementary Appendix Table 5).

## Discussion

### Spatial interaction mechanism of urban expansion and water pollutant emissions

It has become the consensus that urban expansion leads to rapid growth in the intensity and scale of water pollutant emissions and aggravates disturbances to the natural purification process of environment^27,28,51^. A disordered urban expansion process leads to negative effects on the water environment such as structural imbalance, functional degradation and even system collapse of the regional ecological environment^24^. The results of this paper show that the spatial interaction between urban expansion and water pollutant emissions had different mechanisms in local and adjacent counties, ultimately affecting the entire YRD.

The positive interaction in the local county showed that in the process of rapid urbanization, the process of urban and rural population and production factors gathering in a central city and urban area were significant factors. From 2010 to 2015, the urbanization rate of the YRD reached nearly 1% per year. As far as the local county where the urban expansion took place, the increase in urban population and the expansion of high pollution production scale was coupled with the characteristics of heavy industrial structure in the YRD; that is, the proportion of pollution-intensive industries such as printing and dyeing, papermaking, chemical products, non-metallic mineral products and food processing was still high^52^. This inevitably led to the aggravation of the counties’ water pollutant emissions.

Furthermore, the positive interaction of adjacent counties showed that the central county attracted and gathered many people and production factors because of its many development advantages, and this agglomeration created a scale effect^53,54^. That is, through spatial spillover, the central county shared its urban expansion opportunities and construction space with the adjacent counties. With the central county as the core, a regional circle of rapid urban expansion and development was formed, resulting in high water pollutant emissions in the adjacent counties and regional water pollution problems across the entire YRD.

Due to agglomeration and scale effects, the pollutants caused by urban expansion consistently spread from local areas to adjacent areas. Considering the control variables in the SLM, we can infer that the traditional industrial structure of the YRD has not changed the economic development mode of high energy consumption and high pollution at the cost of the environment. The increase in urban population and the acceleration of construction investment are primary manifestations of urban expansion. The local and adjacent counties in the YRD are typically economically similar, which have similar industrial structures, production technologies and capital markets^40,55^. Economic growth leads to the agglomeration of high pollution levels and high-water consumption factors, leading directly to an increase in regional water pollutant emissions. Conversely, the agglomeration of green production factors may lead to a reduction in water pollutant emissions, thus forming a significant spatial spillover effect of water pollution.

However, with improvements of the environmental controls in the YRD, foreign-funded pollution transfer path and pollution haven phenomena have changed. The rising cost of environmental regulation urges local governments to phase out pollution-intensive, foreign-funded enterprises and update production processes to reduce emissions. It is worth noting that other studies have found that improvements in financial autonomy aggravate the local pollution problem at the city level^56,57^. However, the results of this study, using counties as the research unit, support the opposite conclusion. The county-level economy of the YRD was developed and its financial autonomy was higher than that of other regions in China. Local governments may have more power to control the expenditures. Especially under the guidance of the policy of high-quality environmental development, more financial resources are invested in local environmental governance, which reduces regional water pollution emissions.

### Policy implications

Based on the analysis of the water pollutant emissions effect and the interaction mechanism of urban expansion in the YRD, combined with the various interactions of *POP*, *PGDP*, *IS*, *FAI*, *FDI*, *FD* and the other control variables on water pollutant emissions, our policy recommendations are as follows:

- Firstly, pay attention to the strong associated effect and spatial interaction process of urban expansion on local water pollution emissions, reasonably control the scale of urban expansion and improve the urban environmental carrying capacity through the optimization of urban internal spatial structure, such as by urban land function replacement, spatial consolidation of land use and industrial structure adjustment.
- Secondly, strengthen water pollution source control and environmental regulation, decrease water and energy consumption, reduce per capita pollutant emissions, set environmental limitations at the county level and strictly control regional pollution transfer and pollution haven phenomena driven by foreign investment.
- Thirdly, as urban agglomeration and metropolitan areas undertake many urban development functions, strengthen industrial and environmental cooperation between cities, reasonably plan the flow path of production factors between cities, avoid the excessive destruction of resources and environment caused by aggressive competition between cities to alleviate regional pollutant emissions and spatial spillover effects.

### Limitations and future directions

To reveal the water pollution effect and spatial coupling characteristics of urban expansion at various distances, future studies could consider attributes such as left and right bank, upstream and downstream or cross boundary as spatial variables. The latest national census of pollution sources could be used to enrich the panel data and thus permit analysis of the interaction mechanism using a long time series, more regional differentiation and the spatial effect of human production and living activities on the regional water environmental system.

## Conclusion

From 2010 to 2015, the water pollutant emissions in the YRD decreased significantly, with the amounts of COD and NH_3_-N emissions decreasing by 41.36% and 35.54%, respectively. COD and NH_3_-N emission intensity similarly decreased by one grade or more in 61.6% and 55.1%, respectively, of the counties in this study. The pattern of high-intensity emissions in counties changed from a contiguous to a scattered distribution, transferring with urban expansion from the central urban area to the surrounding areas. Coastal and riverside locations attract urban expansion, and water pollution is the most serious within 100 km of the coast and 50 km of the riverside.

Furthermore, the estimated results of the SLM show that the associated effect of urban expansion and water pollutant emissions in the YRD was significant and stable. In 2015, every 1% increase in the *UES* caused increases of 0.297% and 0.335% in COD and NH_3_-N emissions, respectively. In the process of rapid urbanization, large-scale urban expansion promotes the inflow of economic and social factors into cities, expanding the scale of industrial production and living activities, intensifying the high-intensity emissions of domestic and industrial water pollutants throughout the urban agglomeration.

Finally, urban expansion has not only a direct, positive effect on local water pollutant emissions but also an indirect, positive effect on the emissions in adjacent areas through a spillover effect. Every 1% increase in the *UES* increased local COD and NH_3_-N emissions by 0.299% and 0.340%, respectively, and increased the emissions in adjacent counties by 0.068% and 0.084%, respectively. In the heavy industrial structure of the YRD, local urban expansion aggravated water pollutant emissions, and the positive effect on the adjacent counties reveals the agglomeration effect of a central city on the production flow in neighboring areas, which speeds up urban expansion and land construction, which indirectly promotes water pollution emissions in adjacent counties.

## Supplementary Information

Supplementary Tables.

## Data Availability

The datasets used and/or analysed during the current study available from the corresponding author on reasonable request.
