# Supplementary material for: The spatial stress of urban land expansion on the water environment of the Yangtze River Delta in China
Source: Sci Rep. 2022 Oct 11;12:17011. doi: 10.1038/s41598-022-21037-2 (PMC9554182; doi:10.1038/s41598-022-21037-2)
Supplement: Supplementary file 1 — Supplementary Tables. [file 41598_2022_21037_MOESM1_ESM.docx]

**Supplementary Material:**

Appendix Table 1. Pearson correlation analysis and VIF test

| Variables | COD | NH_3_-N | UES | POP | PGDP | | IS | | | FDI | | | FAI | | FD |
| --- | --- | --- | --- | --- | --- | --- | --- | --- | --- | --- | --- | --- | --- | --- | --- |
| UES | 0.503*** | 0.573*** | 1.000 |  |  |  | |  |  |  |  |  |  |  |  |
| POP | 0.679*** | 0.677*** | 0.484*** | 1.000 |  | |  | |  | |  |  |  |  |  |
| PGDP | -0.085** | 0.097** | 0.408*** | -0.175*** | 1.000 | |  | | |  | |  | |  |  |
| IS | 0.095** | -0.062 | 0.135*** | -0.062 | -0.030 | | 1.000 | | |  | | |  | |  |
| FDI | 0.052 | 0.224*** | 0.477*** | 0.166*** | 0.621*** | | -0.123*** | | | 1.000 | | |  | |  |
| FAI | 0.217*** | 0.299*** | 0.664*** | 0.360*** | 0.626*** | | 0.105*** | | | 0.546*** | | | 1.000 | |  |
| FD | 0.015 | 0.187*** | 0.471*** | -0.101** | 0.774*** | | -0.031 | | | 0.556*** | | | 0.533*** | | 1.000 |
| VIF |  |  | 2.49 | 2.25 | 4.23 | | 1.15 | | | 1.96 | | | 3.03 | | 2.80 |

*Note：****P < 0.01, **P < 0.05 and *P < 0.1

Appendix Table 2. Water pollutant emissions in the Yangtze River Delta

| Region | COD | | | | NH_3_-N | | | | | | |
| --- | --- | --- | --- | --- | --- | --- | --- | --- | --- | --- | --- |
|  | total emissions (×10^4^t) | | average county emissions (t) | | | total emissions (×10^4^t) | | average county emissions (t) | | | |
|  | 2010 | 2015 | 2010 | 2015 | | 2010 | 2015 | 2010 | | 2015 | |
| Shanghai City | 92.43 | 49.63 | 8887.86 | 4772.18 | | 10.61 | 5.63 | 1020.58 | 541.79 | |  |
| Jiangsu Province | 119.70 | 74.20 | 12469.25 | 7729.03 | | 15.31 | 10.24 | 1595.20 | 1066.24 | |  |
| Zhejiang Province | 24.26 | 14.75 | 15161.29 | 9219.18 | | 4.74 | 3.84 | 2963.49 | 2397.84 | |  |
| Anhui Province | 78.62 | 46.15 | 8833.74 | 5184.91 | | 11.23 | 7.30 | 1261.50 | 820.30 | |  |
| the YRD | 315.02 | 184.73 | 10328.42 | 6056.58 | | 41.90 | 27.01 | 1373.67 | 885.50 | |  |

Appendix Table 3. Counties exceeding the standard limits for COD and NH_3_-N concentration

| Status | Grading threshold | COD | | NH_3_-N | |
| --- | --- | --- | --- | --- | --- |
|  |  | Percentage of counties exceeding limits | Average CESI | Percentage of counties exceeding limits | Average CESI |
| Exceeding | $R_{ijk}$＞0 | 18.93 | 0.316 | 16.43 | 1.079 |
| Critically exceeding | -0.3＜$R_{ijk}$≤0 | 35.71 | -0.171 | 25.36 | -0.163 |
| Not exceeding | $R_{ijk}$≤-0.3 | 45.36 | -0.521 | 58.21 | -0.644 |
| Total | | 100.00 | -0.238 | 100.00 | -0.244 |

Appendix Table 4. Estimation results of associated effects based on inverse distance matrix

| Variables | COD | | NH_3_-N | |
| --- | --- | --- | --- | --- |
|  | 2010 | 2015 | 2010 | 2015 |
| C | 1.945*** | 2.078** | 0.144 | -1.681* |
| ln USE | 0.327*** | 0.307*** | 0.303*** | 0.347*** |
| ln POP | 0.795*** | 0.653*** | 0.849*** | 0.727*** |
| ln PGDP | 0.011 | 0.159** | 0.104** | 0.344*** |
| ln IS | 0.229*** | -0.020 | -0.037 | -0.187** |
| ln FDI | -0.096*** | -0.181*** | -0.073*** | -0.125*** |
| ln FAI | 0.052 | 0.096 | -0.022 | -0.065 |
| ln FD | -0.059 | -0.149 | 0.092 | -0.048 |
| *ρ* | 0.008* | 0.056*** | 0.031* | 0.051** |
| *Sigma^2* | 0.186 | 0.264 | 0.148 | 0.263 |
| *R^2^* | 0.699 | 0.541 | 0.741 | 0.579 |
| *LLF* | -177.012 | -229.746 | -141.774 | -229.378 |

*Note: ***P < 0.01, **P < 0.05 and *P < 0.1*

Appendix Table 5. Spatial effect decomposition based on inverse distance matrix

| Explanatory variables | COD_2015 | | | NH_3_-N_2015 | | |
| --- | --- | --- | --- | --- | --- | --- |
|  | Direct effect | Indirect effect | Total effect | Direct effect | Indirect effect | total effect |
| ln UES | 0.306*** | 0.005** | 0.311*** | 0.346*** | 0.005** | 0.351*** |
| ln POP | 0.652*** | 0.011*** | 0.663*** | 0.730*** | 0.011** | 0.741*** |
| ln PGDP | 0.158** | 0.003* | 0.160** | 0.347*** | 0.005** | 0.352*** |
| ln IS | -0.022 | 0.000 | -0.022 | -0.186** | -0.003* | -0.189** |
| ln FDI | -0.182*** | -0.003** | -0.185*** | -0.125*** | -0.002** | -0.127*** |
| ln FAI | 0.098 | 0.002 | 0.099 | -0.067 | -0.001 | -0.068 |
| ln FD | -0.150* | -0.003 | -0.153* | -0.048 | -0.001 | -0.049 |

*Note: ***P < 0.01, **P < 0.05 and *P < 0.1*
